# Decoding complex inherited phenotypes in rare disorders: the DECIPHERD initiative for rare undiagnosed diseases in Chile

**DOI:** 10.1038/s41431-023-01523-5

**Published:** 2024-01-04

**Authors:** M. Cecilia Poli, Boris Rebolledo-Jaramillo, Catalina Lagos, Joan Orellana, Gabriela Moreno, Luz M. Martín, Gonzalo Encina, Daniela Böhme, Víctor Faundes, M. Jesús Zavala, Trinidad Hasbún, Sara Fischer, Florencia Brito, Diego Araya, Manuel Lira, Javiera de la Cruz, Camila Astudillo, Guillermo Lay-Son, Carolina Cares, Mariana Aracena, Esteban San Martin, Zeynep Coban-Akdemir, Jennifer E. Posey, James R. Lupski, Gabriela M. Repetto

**Affiliations:** 1 Program for Immunogenetics and Translational Immunology, Institute of Science and Innovation in Medicine, Facultad de Medicina, Clinica Alemana Universidad del Desarrollo, Santiago, Chile; 2 Hospital Dr. Roberto del Río, Santiago, Chile; 3 Rare Diseases Program, Center for Genetics and Genomics, Institute of Science and Innovation in Medicine, Facultad de Medicina, Clínica Alemana Universidad del Desarrollo, Santiago, Chile; 4 Unidad de Gestión Clínica del Niño, Hospital Padre Hurtado, Santiago, Chile; 5 Biosoluciones UDD, Santiago, Chile; 6 Laboratorio de Genética y Enfermedades Metabólicas, Instituto de Nutrición y Tecnología de los Alimentos (INTA), Universidad de Chile, Santiago, Chile; 7 Hospital Base de Valdivia, Valdivia, Chile; 8 Department of Dermatology, Facultad de Medicina Universidad del Desarrollo, Clínica Alemana de Santiago, Vitacura, Chile; 9 Department of Dermatology, Hospital Exequiel González Cortés, Vitacura, Chile; 10 Division of Pediatrics, Facultad de Medicina, Pontificia Universidad Católica de Chile, Santiago, Chile; 11 Genetics Unit, Hospital Dr Luis Calvo Mackenna, Santiago, Chile; 12 Hospital Dr Guillermo Grant Benavente, Concepcion, Chile; 13 University of Texas Health Science Center at Houston, School of Public Health, Department of Epidemiology, Human Genetics and Environmental Sciences, Santiago, Chile; 14 Department of Molecular and Human Genetics, Baylor College of Medicine, Houston, TX USA; 15 Department of Pediatrics, Baylor College of Medicine, Houston, TX USA

**Keywords:** Genetics research, Medical genomics, Disease genetics

## Abstract

Rare diseases affect millions of people worldwide, and most have a genetic etiology. The incorporation of next-generation sequencing into clinical settings, particularly exome and genome sequencing, has resulted in an unprecedented improvement in diagnosis and discovery in the past decade. Nevertheless, these tools are unavailable in many countries, increasing health care gaps between high- and low-and-middle-income countries and prolonging the “diagnostic odyssey” for patients. To advance genomic diagnoses in a setting of limited genomic resources, we developed DECIPHERD, an undiagnosed diseases program in Chile. DECIPHERD was implemented in two phases: training and local development. The training phase relied on international collaboration with Baylor College of Medicine, and the local development was structured as a hybrid model, where clinical and bioinformatics analysis were performed in-house and sequencing outsourced abroad, due to lack of high-throughput equipment in Chile. We describe the implementation process and findings of the first 103 patients. They had heterogeneous phenotypes, including congenital anomalies, intellectual disabilities and/or immune system dysfunction. Patients underwent clinical exome or research exome sequencing, as solo cases or with parents using a trio design. We identified pathogenic, likely pathogenic or variants of unknown significance in genes related to the patients´ phenotypes in 47 (45.6%) of them. Half were de novo informative variants, and half of the identified variants have not been previously reported in public databases. DECIPHERD ended the diagnostic odyssey for many participants. This hybrid strategy may be useful for settings of similarly limited genomic resources and lead to discoveries in understudied populations.

## Introduction

Rare diseases (RD) affect all populations worldwide. Although each disease is individually rare, their cumulative prevalence is estimated at approximately 5% of the population [1]. With a global population of nearly 8 billion people in 2023, this figure suggests that around 400 million people may suffer from an RD, and 330 million of them could live in low-and-middle-income countries (LMIC) (estimated from [2]). Given the rarity of each condition, affected individuals, families and health systems face many challenges in access and quality of care. The prolonged “diagnostic odyssey” or “medical pilgrimage” is among these challenges, which can last decades [3].

The majority of RDs have a genetic etiology. Exome and genome sequencing (ES and GS, respectively) have revolutionized diagnosis and discovery of RDs in the past 15 years in an unprecedented way [4], particularly for persons with rare undiagnosed disorders (RUD). The early use of ES and GS has been shown to be cost-effective compared with stepwise testing, and has clinical utility in terms of changes in clinical management and reproductive decisions [5–10]. Nevertheless, these powerful diagnostic tools are not available globally. Only 31 countries have ES as a clinical diagnostic service listed in the Genetic Testing Registry [11], and most of them are in high- or high-middle-income countries, according to World Bank classification, except for India and Bangladesh, which have large pópulations sizes and thus likely high testing volumes [12]. The lack of availability and coverage of complex laboratory genomic services increases the already substantial healthcare gaps for persons with RD. Barriers to implementation of ES and GS in LMICs include lack of adequately trained and certified personnel, especially in laboratory genetics and genomics and in bioinformatics; higher costs of equipment and reagents compared with the global North, lack of coverage and reimbursement of testing, and insufficient regulatory, legal and ethics frameworks [13, 14]. As a result, there is limited availability of genomic testing for persons with RDs, and therefore, prolonged, and unsolved diagnostic odysseys. In some countries, patients and clinicians opt to outsource testing, but this usually requires substantial personal out-of-pocket payments, which also limits access to only those who can afford these payments [15, 16].

Although Chile is a high-income country according to World Bank standards [12], it can be considered as an “emerging nation” in terms of genomic medicine implementation [17]. For example, to date, there are no high-throughput sequencers in clinical laboratories to provide ES or GS for diagnosis of RD in a cost- and time-efficient manner, and there are no training or certified programs in clinical laboratory genetics and genomics or in bioinformatics.

It remains unknown if and how countries with limited genomic resources can develop RD diagnostic resources. To overcome this gap, we implemented a program for RD in Chile, called DEcoding Complex Inherited PHenotypes of Rare Diseases (DECIPHERD), with a specific focus on patients with RUD. The strategy was implemented in two phases: first, a training phase, which relied on international collaboration, followed by a second, local development phase. This second phase used a hybrid strategy, with clinical evaluation and variant data analysis interpretation performed locally, but ES outsourced abroad. We report on the process of implementation, and the results of the first 103 analyzed probands.

## Methods

Implementation of the DECIPHERD sequencing and analysis pipeline consisted of two phases: a training phase and a local development phase. During the training phase, samples from probands and their parents were submitted to Baylor College of Medicine (BCM) for trio ES. The resulting exome data was analyzed by members of the DECIPHERD team who had the opportunity to train at BCM. In addition, a workshop on variant interpretation, led by collaborators from the US, was hosted at Universidad del Desarrollo (UDD). In parallel, probands underwent clinical exome sequencing (CES) and analysis at UDD. Comparisons of the overall diagnostic yield of both strategies and of the concordance of results for a subset of probands that were analyzed under both strategies were performed to decide the best design for the local development phase.

### Participants

Probands with RUD, of any age or sex, and residing in Chile, were candidates to participate. The inclusion criteria were: [1] the presence of 2 or more major congenital anomalies, or one major and several minor anomalies, [2] neurological abnormalities such as global developmental delay or intellectual disability with one major or multiple minor congenital anomalies, or [3] significant dysfunction of the immune system, for which a genetic etiology was suspected. Probands were referred to the DECIPHERD study by their treating physicians if a cause was not identified despite prior evaluations at their local health centers (e.g., karyotype, FMR1, microarray, metabolic or gene panel testing, among others). Demographic information was obtained from interviews with probands and/or parents or guardians. Candidate participants were presented by their treating physicians at a weekly screening meeting led by the core DECIPHERD team, which includes clinicians, laboratory geneticists and bioinformaticians. Clinical history was presented using a structured form, and the probands’ clinical features were described using Human Phenotype Ontology (HPO) terms [18]. After a multidisciplinary discussion, probands that fulfilled the inclusion criteria were invited to participate. Parent participation for trio analysis was preferred when possible. Written informed consent was obtained from the participants and/or their guardians.

### Samples and DNA extraction

Blood samples were obtained in EDTA tubes from probands and their parents when available. Genomic DNA was extracted using the DNeasy Blood and Tissue Kit (Qiagen, Hilden, Germany).

### Exome sequencing and bioinformatics pipelines

#### Training phase

For trio exome sequencing at BCM (BCM-ES-trio), exome enrichment libraries were obtained using the NimbleGen VCRome 2.1 kit (Roche, Switzerland) according to the manufacturer’s protocol. These samples were subsequently sequenced using Illumina HiSeq 2500 equipment (Illumina, CA, USA). All data was aligned to GRCh37. Data processing and variant annotation was performed using the Variant Analyzer bioinformatic pipeline developed at BCM (https://github.com/BCM-Lupskilab/VariantAnalyzer). Variants with less than 3 reads were excluded, as well as synonymous, deep intronic and intergenic variants, and 3’ and 5’ UTR regions. Annotated variants were evaluated using both recessive, dominant, and X-linked models. For the recessive model, variants with minor allele frequency (MAF) greater than 0.005 within the Baylor-Hopkins Center for Mendelian Genomics (CMG), ESP 5400, 1000 Genomes, gnomAD v.3 and ExAC databases were excluded, as well as in our internal database control population database (unpublished). Also, potential recessive variants were excluded if they were present in the ExAC database with a homozygous or hemizygous count of 10 or greater. For autosomal dominant model analyses, variants were excluded if their MAF were greater than 0.001 in these databases, and if they were present in the gnomAD V3 and our database with an allele count greater than 5. Rare variants were analyzed manually considering specific variant characteristics including type of variant and the combined annotation dependent depletion (CADD) score (*)*. Different publicly available resources were used to evaluate the association of a patient’s phenotype with his/her detected variant allele such as OMIM (https://www.omim.org), gnomAD (https://gnomad.broadinstitute.org), UCSC Genome browser (https://genome.ucsc.edu), PubMed (https://pubmed.ncbi.nlm.nih.gov/*)* among others.

A subset of candidate variants was orthogonally confirmed by Sanger sequencing at UDD. Regions containing the genetic variants were amplified by PCR with custom-design primers using the ApE software [19] (ApE-A plasmid Editor (utah.edu) and synthetized by Macrogen™ (Korea)). The resulting ~300 bp amplicons were sequenced bidirectionally using BigDye™ Terminator v1.1 Cycle Sequencing Kit (Applied Biosystems, Foster City, CA, US) and the SeqStudio™ Genetic Analyzer (Thermo Fischer Scientific, Waltham, MA, US).

#### Local development phase

In parallel, and due to the lack of available high-throughput sequencing equipment in clinical laboratories in Chile, we initially performed clinical exome sequencing (CES) in probands only at UDD (UDD-CES-Solo), using the SOPHiA Clinical Exome Solution V1 (SOPHiA Genetics, Lausanne, Switzerland). This method captures exons from 4964 genes known to cause Mendelian disorders. Sequencing was carried out in an IIlumina MiSeq sequencer. Results were analyzed using the SOPHiA DDM™ software with the same filters described above and compared with those obtained in the training phase. A subset of candidate variants was confirmed by Sanger sequencing in the proband and parents if available as described above.

Subsequently, we opted for a hybrid model, where ES was performed abroad, and the analysis of the results was performed at UDD by laboratory scientists of the DECIPHERD core team (DECIPHERD-ES). Sequencing was outsourced to Novogene (Beijing, China) using SOPHiA Exome Solution_V1 capture (SOPHiA Genetics, Lausanne, Switzerland), that captures the exons of approximately 19,680 protein-coding genes. The resulting annotated variants were evaluated by the core DECIPHERD team using the SOPHiA DDM™ platform. This platform provides information on population frequencies of the databases mentioned above, but it also includes those obtained from the SOPHIA users´ community and our local cohort. All the annotated variants were reviewed stepwise according to their inheritance model, starting by autosomal recessive (AR, considering homozygous and compound heterozygous variants), followed by autosomal dominant (AD) and X-linked (XL) models, as well as de novo occurrences, using the same frequency filters described for the training phase, and with the additional filter of excluding variants present in two or more unrelated participants from our cohort. The process also allows the detection of copy-number variants (CNVs). HPO terms were used to create virtual gene panels to initially narrow down the search for candidate variants within genes already associated to each patient´s phenotype, subsequently the exomes were also analyzed in a phenotypically unbiased manner. Rare variants were analyzed manually considering variant characteristics including type of mutation and combined annotation dependent depletion (CADD) score. The same databases described above were used to obtain additional variant information. We then prioritized the filtered variants based on their ACMG interpretation provided by the SOPHiA platform and further assessed their interpretation with additional freely available resources, including ClinVar (www.ncbi.nlm.nih.gov/clinvar), Varsome (www.varsome.com) and Franklin (https://franklin.genoox.com). Candidate variants were interpreted as either pathogenic (P), likely pathogenic (LP), variant of unknown significance (VUS), likely benign (LB) or benign (B). In case of discrepancies among these resources, the interpretation provided by ClinVar, if available, was selected; if a variant was not included in ClinVar, the most common interpretation among the other resources was chosen.

### Clinical interpretation

Results were considered “informative” when a P, LP variant or VUS was identified in a gene consistent with the proband’s phenotype. Informative cases were then labeled as “solved” for P/LP variants and “suggestive” when a VUS was identified. Recessive conditions in which only one informative variant was identified were considered “partially informative”, given that we could not define if this represented carrier status only, a novel potentially dominant form of the disease, as has been previously described for other disorders [20] or a compound heterozygote for a non-coding variant undetected by this method. Results were considered “uninformative” when there were no variants identified that could explain the patient’s phenotype. Results were returned to families and referring clinicians in a genetic counseling session and with a written report.

### Statistical analysis

All data were stored in REDCap [21]. Descriptive analysis included frequencies, means and ranges. To identify variables associated with the likelihood of obtaining an informative result, first, we built bivariate logistic regression models for each of the following variables: sex, age (in years, and categorized in groups representing childhood (0–12 years), adolescence (12–18 years) and adulthood (18–52 years)), type of care (public or private), number of systems affected (based on the grouping of individual HPO terms into “systems” according to their hierarchy in the HPO’s terms tree), and whether the patient had congenital anomalies, neurological or immune compromise (represented as binary variables each). Then, we built a multivariate regression model including all variables mentioned before. The analyses were performed in RStudio v.2023.06.0 + 421 with R v.4.2.2, using the glm function and the binomial family modifier.

## Results

### Demographic and clinical characterization of patients

Between June 2019 and December 2022, 133 probands were screened for the study and 103 of them consented to participate. Non-participants included probands who did not fulfill inclusion criteria (*n* = 21) or declined to participate (*n* = 9).

**Fig. 1:**
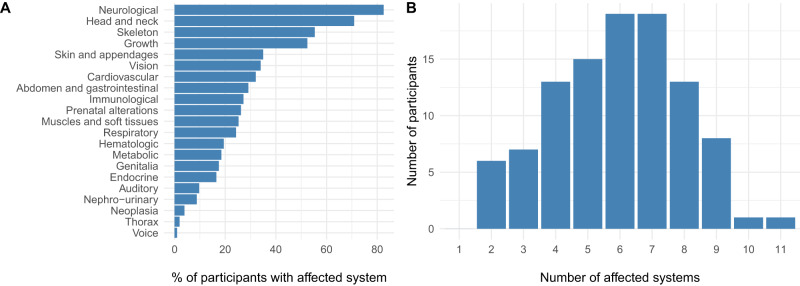
Clinical manifestations. The proband’s phenotypes were categorized into systems based on the Human Phenotype Ontology (HPO) hierarchy tree. **A** Frequency of affected systems in probands. The figure shows the percentage of probands with the corresponding system affected. **B** Number of affected systems per proband. Absolute frequency of systems affected per participant, based on the previously mentioned definition of system.

To characterize the probands’ phenotypes, we collected information from clinical records and in-person evaluations, describing clinical manifestations using HPO terms. Demographic characteristics are summarized in Table 1. The most frequent clinical inclusion criteria were congenital anomalies, with or without neurological abnormalities, present in 75% of probands (Supplementary Table 1). When grouped by systems, the overall cohort had involvement of 21 different organ systems. The majority of probands had neurologic manifestations, followed by craniofacial, musculoskeletal and growth manifestations (Fig. 1a). All probands had manifestations in at least two major systems, with a median of six and a range of two to eleven compromised systems (Fig. 1b). These results show that probands had heterogeneous and complex clinical manifestations.

**Table 1:** Demographic characterization of probands.

		Age (yrs) at recruitment	Public health care	Health care provider located in Santiago
Sex	*N* (%)	Median (IQR) [range]	*N* (%)	*N* (%)
Male	49 (47.6)	8 (11) [0–52]	39 (79.6)	43 (48.9)
Female	54 (52.4)	9 (14) [0–44]	35 (64.8)	45 (52.1)
Total	103 (100)	8 (13) [0–52]	74 (71.8)	88 (85.4)

### Comparison of findings in the training and initial development phase

During the training and the initial development phases, 13 trios had BCM-ES and 16 probands had UDD-CES, with an overlap of 6 probands. An informative finding was identified in 7 BCM-ES-trio analysis (53.8%) and 4 UDD-CES-solo analysis (25%). Three of the six overlapping probands had informative findings identified by both strategies. In addition, one UDD-CES-solo uninformative case had an informative result by BCM-ES-trio, resulting from a gene that was not included in the CES panel. The higher yield of trio ES compared with CES led to the decision to continue with ES in the local development phase.

### Exome analysis

After the learning phase, 74 additional probands were included in the local development phase (DECIPHERD-ES) using the hybrid model. From this point onward, findings are presented in aggregated form including both the training and local development phases. Ten probands with uninformative results on UDD-CES subsequently had DECIPHERD-ES. Therefore, we performed 113 total analyses in the 103 probands, of which 97 were ES. Of those undergoing ES, seventy-one (69%) participated as trios with both parents, 7 (6.7%) as duos (proband with mother, 3.8%) and 19 were solo cases (19.6%), for a total of 246 participants.

Forty-seven probands (45.6%) had informative results, that is a P/LP variant or a VUS in genes known to cause conditions consistent with their clinical phenotypes. The different strategies had different yields of informative results. CES provided informative results for 4 of 16 probands (25%), ES-solo for 9 of 19 (47.5%), ES duo in 2 of 7 (28.6%) and ES-trio in 32 of 71 families (45.0%). The results of these strategies are summarized in Table 2.

**Table 2:** Detection rate by familial and sequencing strategies.

Strategy	Number of analyses	Number of informative variants	Probands with informative findings, *N* (%)
CES	16	4	4 (25.0)
ES	97	51*	43 (44.3)
ES-Solo	19	9	9 (47.3)
ES-Duo	7	2	2 (28.6)
ES-Trio	71	40	32 (45.0)
Total	113^a^	55	47 (45.6)

^a^Ten uninformative CES were resequenced as ES-Solo, for a total of 113 analyses in the 103 patients.

*Eight probands had two distinct variants each: three in two different genes/regions, and five as compound heterozygotes.

Thirty-one of these 47 probands with informative results were considered “solved” cases, i.e. a molecular diagnosis concluded with the identification of P/LP variants (30% of all probands and 66% of those with informative results), of which 23 had an AD condition, 4 AR and 4 XL. In contrast, 13 cases had VUS and were considered as “suggestive results” (12.6% of all probands and 27.6% of those with informative results, respectively), of which 6 corresponded to AD conditions, 3 AR and 4XL. Finally, three cases were considered “partially informative” (2.9% and 6.4%, respectively) since they had a single variant identified in a gene associated with a recessive condition and a phenotype consistent with this AR disease. Clinical and molecular findings of the patients with informative results can be found in Table 3.

**Table 3:** Informative variants.

	Patient ID	Age at inclusion in the study (years/months)	Sex	Referral phenotypes	Transcript	Gene variant	Protein variant	ClinVar ID	Zygosity	Inheritance	Variant Interpretation*	OMIM Phenotype (^a^ID)	OMIM Phenotype Inheritance
Variant found in ClinVar	3	2	M	DD; severe failure to thrive; aortic root dilation; micropenis; DFF	—	seq[GRCh37]chrX:69746790-80556046dup	—	154251	Hem.	Mat.	P	Duplication Xq13q21	XLR
	4	4	F	DD; speech delay; labia anomalies; limb anomalies; DFF. Affected sibling and mother	NM_001031804	MAF:c.185 C > T	p.(Thr62Met)	1320073	Het.	Mat. (suspected^a^)	LP	Ayme-Gripp Syndrome (#601088)	AD
	17	15	F	ID; ASD; microcephaly; strabismus; gastroesophageal reflux; limb anomalies	NM_006766.5	KAT6A:c.3385 C > T	p.(Arg1129*)	180229	Het.	De Novo	P	Arboleda-Tham Syndrome (#616268)	AD
	18 (b)	10	F	ID; ISD Juvenile idiopathic arthritis; behavioral abnormality; DFF; parental consanguinity; deceased sibling	NM_014844.4	TECPR2:c.4006 C > T	p.(Arg1336Trp)	996005	Hom.	Mat.; Pat.	VUS	Neuropathy, hereditary sensory and autonomic, type IX, with developmental delay (#615031)	AR
	24	17	F	ID; microcephaly; microphthalmia; short stature; DFF; sibling with similar phenotype, deceased at 1 week of age	NM_001270449	UBE3B:c.61 G > T	p.(Glu21*)	225041	Hom.	Mat.; Pat.	P	Kaufman Oculocerebrofacial Syndrome (#244450)	AR
	27	10 m	M	DD; macrocephaly; seizure; hypotonia; DFF; Mother had 2 miscarriages/stillborn with anencephaly	NM_001083962	TCF4:c.1165 C > T	p.(Arg389Cys)	1319340	Het.	De Novo	LP	Pitt Hopkins Syndrome (#610954)	AD
	32	20	M	ID; microcephaly; minor facial anomalies; scoliosis; renal tubular acidosis; poikiloderma	NM_001029882	AHDC1:c.3814 C > T	p.(Arg1272*)	431102	Het.	De Novo	P	Xia-Gibbs Syndrome (#615829)	AD
	35	2 m	F	Cerebellar vermis hypoplasia; perimembranous ventricular septal defect; oro-pharyngeal dysphagia	—	seq[GRCh37]chr6:148682004-155706788del	—	150766	Het.	De Novo	P	Deletion 6q24q25 (#612863)	AD
	48	1 m	M	Polyhydramnios; decreased fetal movement; seizures. Deceased. Two affected siblings, deceased.	NM_020686	ABAT:c.168+1 G > A	p.(?)	635252	Hom.	Mat.; Pat.	P	GABA Transaminase Deficiency (#613163)	AR
	64	13	F	Polycystic kidney dysplasia; recurrent infections; ISD (sepsis)	NM_001009944.2	PKD1:c.8300 G > A	p.(Arg2767His)	1256450	Het.	Pat.	VUS	Polycystic Kidney Disease 1 (#173900)	AD
						PKD1:c.8291 T > C	p.(Met2764Thr)	976837	Het.	Mat.	VUS		
	86	5	M	DD; microcephaly; severe short stature; strabismus; cryptorchidism; clinodactyly of the 5th finger; DFF	NM_004187.3	KDM5C:c.860 C > T	p.(Ser287Leu)	945345	Hem.	Mat.	VUS	Intellectual developmental disorder, X-linked syndromic, Claes-Jensen type (#300534)	XLR
	87	23	M	ID; borderline; ASD; macrocephaly; cranial hyperostosis; myopia; astigmatism; obesity	NM_001110792.1	MECP2:c.724 C > A	p.(Pro242Thr)	1439710	Hem.	Mat.	VUS	Intellectual Developmental Disorder, X-linked, syndromic type 13 (#300055)	XLR
	95	15	M	ID; seizures; microcephaly; short stature; VSD; micropenis; DFF	NM_032119.3	ADGRV1:c.6994 A > T	p.(Ile2332Phe)	46359	Het.	Pat.	VUS	Usher Syndrome, Type IIc; USH2C (#605472)	AR, DD
						ADGRV1:c.17461 T > C	p.(Ser5821Pro)	1915342	Het.	Mat.	VUS		
	96	18	F	ID-profound; conductive hearing impairment; short stature; DFF	NM_004586.2	RPS6KA3:c.1269 A > T	p.(Glu423Asp)	1348599	Het.	Mat.	VUS	Mental Retardation, X-linked 19; Coffin-Lowry Syndrome (#300844; #303600)	XLD
	107	12	F	Motor delay; hearing impairment; strabismus; laryngomalacia; umbilical hernia; skeletal anomalies; DFF	NM_003036	SKI:c.101 G > T	p.(Gly34Val)	39783	Het.	Unknown	P	Shprintzen-Goldberg Craniosynostosis Syndrome (#182212)	AD
	112	11	M	ID; seizure; hypertonia; ASD; DFF	—	seq[GRCh37]chr15:22873285-28544664del	—	146597	Het.	De Novo	P	Angelman Syndrome (#105830)	AD
	115	23	M	ID; macrocephaly; short stature; tooth agenesis; cryptorchidism; skeletal anomalies; DFF	NM_001164342.2	ZBTB20:c.1805G>A	p.(Gly602Asp)	684507	Het.	De Novo	VUS	Primrose Syndrome (#259050)	AD
	132	15	F	ID-severe; seizure; feeding difficulties; short stature; scoliosis	NM_007327	GRIN1:c.1852G>C	p.(Gly618Arg)	981271	Het.	Unknown	LP	Neurodevelopmental Disorder With Or Without Hyperkinetic Movements And Seizures, Autosomal Dominant (#614254)	AD
Variant not found in ClinVar	1	8	F	ID; brain structural alterations; sensorineural hearing impairment; optic atrophy; constipation; DFF	NM_001356.4	DDX3X:c.1045 G > A	p.(Ala349Thr)	—	Het.	De Novo	LP	Intellectual Developmental Disorder. X-linked. Syndromic. Snijders Blok Type (#300958)	XLD. XLR
	2	25	M	ID; ASD; macrocephaly; minor facial anomalies; myopia; limbs anomalies; scoliosis	NM_152641.4	ARID2:c.1746_1747delAG	p.(Arg582SerfsTer6)	—	Het.	De Novo	LP	Coffin-Siris Syndrome 6 (#617808)	AD
	5	19	F	ID; microcephaly; short stature; horseshoe kidney; skeletal anomalies; DFF	NM_144572	TBC1D2B:c.274 C > T	p.(Gln92*)	—	Hom.	Mat.; Pat.	LP	Neurodevelopmental Disorder With Seizures And Gingival Overgrowth (#619323)	AR
	26	13	M	DD; tall stature; abnormal aggressive, impulsive or violent behavior; strabismus; skeletal anomalies; DFF	NM_002501	NFIX:c.274delG	p.(Val92Cysfs*2)	—	Het.	De Novo	LP	Malan Syndrome (#614753)	AD
	30 (c)	2	F	DD; short stature; iris coloboma; chorioretinal coloboma; minor limb anomalies; pineoblastoma; DFF	NM_014281	PUF60:c.1574 T > A	p.(Val525Glu)	—	Het.	De Novo	LP	Verheij Syndrome (#615583)	AD
	45	6	F	Global DD; seizure; craniosynostosis; short stature; strabismus; DFF;	NM_032436.3	CHAMP1:c.1903_1906del	p.(Glu635Thrfs*2)	—	Het.	De Novo	LP	Mental retardation. autosomal dominant 40 (#616579)	AD
	57	19	M	Hypogonadotropic hypogonadism; optic nerve hypoplasia	NM_006941	SOX10:c.341 G > C	p.(Trp114Ser)	—	Het.	Unknown	LP	Waardenburg Syndrome TYPE 4 C (#613266)	AD
	62	7	F	Global DD; microcephaly; cerebellar atrophy; gait ataxia	NM_006946.2	SPTBN2:c.185 C > T	p.(Thr62lle)	—	Het.	De Novo	VUS	Spinocerebellar Ataxia 5 (#600224)	AD
	65	6	M	Conductive hearing impairment; strabismus; hypermetropia; cutaneous finger syndactyly; cryptorchidism; DFF. Affected father	NM_005450.4	NOG:c.553 T > C	p.(Ser185Pro)	—	Het.	Pat.	LP	Stapes ankylosis with broad thumbs and toes (#184460)	AD
	67	7	F	Global DD; speech delay; hypotonia; microcephaly; abnormal heart morphology; skeletal anomalies; DFF	NM_004247	EFTUD2:c.766_767del	p.(Cys256Hisfs*5)	—	Het.	De Novo	LP	Mandibulofacial Dysostosis. Guion-almeida Type (#610536)	AD
	71	7	M	DD; dextrocardia; congenital diaphragmatic hernia; strabismus; ISD (recurrent otitis); hirsutism	NM_001128844.1	SMARCA4:c.3712 T > G	p.(Ser1238Ala)	—	Het.	De Novo	LP	Coffin-Siris Syndrome type 4 (#614609)	AD
	81	15	M	ID; seizure; agenesia of corpus callosum; hypotonia; atrial septal defect; cryptorchidism; joint hypermobility	NM_001193465	KANSL1:c.2692 A > T	p.(Lys898*)	—	Het.	De Novo	LP	Koolen-de Vries Syndrome (#610443)	AD
	82	5 m	F	Clinodactyly; ventricular septal defect; abnormality of the anus; premature birth; DFF	NM_006496	GNAI3:c.136 A > G	p.(Lys46Glu)	—	Het.	De Novo	LP	Auriculocondylar Syndrome 1 (#602483)	AD
	85	12	F	Cataracts; 2-3 toe syndactyly; ISD (recurrent otitis media); precocious puberty; DFF	NM_001123385	BCOR:c.4799dupT	p.(Tyr1601Leufs*7)	—	Het.	De Novo	LP	Microphthalmia. Syndromic 2/ Oculofaciocardiodental Syndrome (#300166)	XLD
	88	12	M	ID severe; macrocephaly; ASD; spastic diplegia; strabismus; DFF	NM_020987	ANK3:c.3380 C > T	p.(Thr1127Met)	—	Het.	De Novo	VUS	Mental Retardation. Autosomal Recessive 37 (#615493)	AR
	90	1	F	DD; retinitis pigmentosa; hypotonia; hypoplasia of the corpus callosum; short stature; ISD (sepsis)	NM_005660	SLC35A2:c.918_929dup	p.(Leu307_Val310dup)	—	Het.	De Novo	VUS	Congenital Disorder Of Glycosylation. Type IIm (#300896)	XLD
	91	3	F	ID; hypotonia; cerebral atrophy; feeding difficulties; umbilical hernia; DFF; parental consanguinity	NM_001080517.2	SETD5:c.3196-2 A > G	p.(?)	—	Hom.	Mat.; Pat.	LP	Mental Retardation. Autosomal Dominant 23 (#615761)	AD
	100	16	F	ID-moderate; seizure; ASD; aggressive behavior; short stature	NM_006035	CDC42BPB:c.2726+1 G > A	p.(?)	—	Het.	De Novo	LP	Chilton-Okur-Chung Neurodevelopmental Syndrome (#619841)	AD
	104	6	M	ID; laryngomalacia; short stature; ventricular septal defect	NM_001197104	KMT2A:c.5874_5878del	p.(Phe1959Valfs*12)	—	Het.	De Novo	LP	Wiedemann-Steiner Syndrome (#605130)	AD
	113	8	F	ID; absent speech; ASD; short stature; sacral dimple; DFF	NM_001347721.1	DYRK1A:c.948_949insGG	p.(Phe317Glyfs*43)	—	Het.	Unknown*	LP	Intellectual developmental disorder. autosomal dominant 7 (#614104)	AD
	116	2	F	Global DD; ASD; absent speech; tall stature; macrocephaly; insomnia; tachycardia; obesity	NM_001099402	CCNK:c.878 C > T	p.(Pro293Leu)	—	Het.	De Novo	VUS	Intellectual Developmental Disorder With Hypertelorism And Distinctive Facies (#618147)	AD
	120	12	F	ID-moderate; short stature; hyperbilirubinemia; acanthosis nigricans; precocious puberty; parental consanguinity	NM_001080453.2	INTS1:c.3010 C > T	p.(Arg1004Trp)	—	Hom.	Mat.; Pat.	VUS	Neurodevelopmental Disorder With Cataracts. Poor Growth. And Dysmorphic Facies (#618571)	AR
	133	3	F	Microcephaly; seizure; agenesis of corpus callosum; short stature; unilateral renal agenesis; VSD; DFF	NM_000284	PDHA1:c.1149 G > A	p.(Trp383*)	—	Het.	De Novo	LP	Pyruvate Dehydrogenase E1-alpha Deficiency (#312170)	XLD
Mixed novelty variants	13	3	F	Neurodegeneration; necrotizing myopathy; metabolic alterations (CK 4000; mildly elevated lactate)	NM_001271934	TK2:c.176 C > T	p.(Thr59Met)	12710	Het.	Mat.	P	Progressive External Ophthalmoplegia With Mitochondrial DNA Deletions, Autosomal Recessive 3 (#617069)	AR
					NM_004614	TK2:c.125_127del	p.(Asp42_Lys43delinsGlu)	—	Het.	Pat.	VUS		
	98	7	M	ID; microcephaly; strabismus; anal atresia; inguinal hernia; cryptorchidism; DFF	NM_005515	MNX1:c.355 C > A	p.(Pro119Thr)	—	Het.	De Novo	VUS	Currarino Syndrome (#176450)	AD
					—	seq[GRCh37]chr22:21738555-22899302del	—	625626	Het.	De Novo	P	Chromosome 22q11.2 deletion syndrome, distal (#611867)	AD
	106	31	F	Short stature; polycystic ovaries; reduced subcutaneous adipose tissue; hearing impairment; scoliosis; DFF	NM_004991.3	MECOM:c.3383 G > A	p.(Cys1128Tyr)	2077252	Het.	Mat.	VUS	Radioulnar Synostosis With Amegakaryocytic Thrombocytopenia 2 (#616738)	AD
					NM_001753.4	CAV1:c.284 C > T	p.(Thr95Met)	—	Het.	Pat.	VUS	Lipodystrophy. Familial Partial. Type 7 (#606721)	AD
	119	1 m	M	Microcephaly; cerebellar hypoplasia; optic nerve hypoplasia; supernumerary nipple; WPW syndrome; VSD; DFF	NM_033026.5	PCLO:c.12814 C > T	p.(Arg4272Cys)	1378579	Het.	Pat.	VUS	Pontocerebellar hypoplasia, type 3 (#608027)	AR
					NM_033026.5	PCLO:c.1656G>T	p.(Gln552His)	—	Het.	Mat.	VUS		
	124	2	F	Global DD; acquired microcephaly; short stature; abnormal foot morphology	NM_000124	ERCC6:c.2203 C > T	p.(Arg735*)	1701	Hem.	Pat.	P	Cockayne Syndrome, Type B (#133540)	AR
					—	seq[GRCh37]chr10:49383876-52383915del	—	—	Het.	Mat.	P		
	125	9 m	F	Global DD; hypotonia; myopathy; DFF	NM_004525.2	LRP2:c.11503 C > T	p.(Arg3835Cys)	1469182	Hom.	Mat.; Pat.	VUS	Donnai-Barrow Syndrome (#222448)	AR
					NM_001267550.2	TTN:c.30503 C > A	p.(Thr10168Lys)	—	Hom.	Mat.; Pat.	VUS	Muscular Dystrophy. Limb-girdle. Autosomal Recessive 10 (#608807)	

The informative variants were organized according to their presence or absence in ClinVar. For patients with mixed novelty variants, the second variant is absent in Clin Var.

*DD* developmental delay, *DFF* dysmorphic facial features, *ID* intellectual disability, *ASD* autism spectrum disorder, *ISD* immune system dysfunction, *VSD* ventricular septal defects, *WPW* syndrome: Wolff-Parkinson-White syndrome, *Hem* hemizygous, *Het* heterozygous, *Hom* homozygous, *Mat* maternal, *Pat* paternal, *Unk* unknown, *AD* autosomal dominant, *AR* autosomal recessive, *XLD* X-linked dominant, *XLR* X-linked recessive, *DD* digenic dominant

*Variant interpretation as provided by ClinVar if available; if not, by the most common interpretation among SOPHiA DDM, Franklin and Varsome.

^a^Mother with similar phenotype, but unavailable for the study. *P* pathogenic, *LP* likely pathogenic, *VUS* variant of unknown significance.

^b^Previously reported in Hum Mutat. 2021 Jun;42(6):762–776. 10.1002/humu.24206.

^c^Previously reported in Am J Med Genet A. 2023 Jun. 10.1002/ajmg.a.63313.

Four patients with suggestive results warrant further description since they illustrate interesting findings. Patient ID#64 had diagnosis of renal and pancreatic cysts at age 2 years, with recurrent infections and was found to have two VUS in PKD1, inherited in trans from healthy parents. This is generally considered an AD disease; however, cases have been described with biallelic hypomorphic mutations in this gene, postulated to be an AR form of disease [22]. Another patient (ID#91) with severe ID and born to consanguineous parents had a homozygous P/LP variant in SETD5, known to cause dominant ID, but with incomplete penetrance and variable expressivity [23, 24]. In this case, one parent was described as having mild ID, and we propose the existence of a potentially more severe recessive phenotype for this condition. Finally, two patients (ID#106 and #125) had VUS in two different genes each, with each variant potentially associated with different components of their phenotype, suggesting that these situations may constitute dual genetic diagnoses.

This heterogenous cohort allowed the identification of novel variants potentially associated with disease. We found 55 distinct informative variants in the 47 probands (considering homozygotes as having one same variant). Fifty variants were SNVs and 5 were CNVs. Among informative variants, 25 (45.4%) were confirmed de novo, and 26 (47.2%) were inherited, underscoring the utility of performing trio ES. Parental samples were unavailable for 4 probands, and thus the inheritance of their variants could not be determined. Twenty-six informative variants (47.3%) were listed in ClinVar as of June 30, 2023. Interestingly, the other twenty-nine informative variants (52.7%) were not listed in ClinVar, of which sixteen were predicted as P/LP and twelve as VUS according to ACMG criteria. In summary, more than half of informative variants had not been reported in public databases at the time of this writing.

Finally, to assess for factors associated with higher likelihood of informative findings, we performed bivariate and multivariate analysis of probands´ demographics and clinical phenotypes using logistic regression. In the bivariate analyses, the presence of neurologic abnormalities, immune dysfunction, and being in the 12–18 years of age group were associated with significant diagnostic yield. In the multivariate analysis, only being in the 12–18 years of age group was positively associated with significant diagnostic yield (Table 4).

**Table 4:** Logistic regression analysis. Identification of variables associated with obtaining an informative result.

	Bivariate analysis			Multivariate analysis		
Variable	OR	[95%CI]	*p*	OR	[95%CI]	*p*
Sex = Male	0.59	[0.27. 1.29]	0.18	0.78	[0.32. 1.88]	0.58
Age (yrs)	0.99	[0.96. 1.03]	0.76	0.93	[0.85. 1.03]	0.15
12–18	3.53	[1.01. 12.38]	0.05*	12.64	[2.05. 78.04]	0.01*
18–52	1.03	[0.37. 2.87]	0.96	3.87	[0.42. 35.3]	0.23
Private health care	1.41	[0.59. 3.33]	0.44	1.13	[0.42. 3.06]	0.80
Neurological compromise	2.73	[1.11. 6.74]	0.03*	2.33	[0.82. 6.58]	0.11
Immune compromise	0.21	[0.07. 0.69]	0.01*	0.25	[0.06. 1.04]	0.06
Congenital anomalies	3.19	[0.82. 12.36]	0.09	1.95	[0.31. 12.3]	0.48
Number of systems compromised	1.11	[0.91. 1.35]	0.30	1.19	[0.94. 1.49]	0.14

*p <= 0.05.

## Discussion

This is the first reported experience of a local implementation program for the genetic evaluation of patients with RUD in Chile using exome sequencing. The process allowed us to establish a trained team for clinical assessment and exome analysis through a rigorous pipeline that enabled the diagnosis in nearly half of the study participants. It also permitted tens of patients with severe previously undiagnosed disorders to receive information on the cause of their condition.

The process of implementation highlights the relevance of international collaboration to obtain critical knowledge and training when these resources are unavailable locally. To circumvent the lack of genomic capabilities at scale in Chile, our strategy relied on outsourcing the sequencing component, while leveraging and developing capacities in the clinical evaluation and bioinformatics analysis that can be performed in-house. Albeit the use of a commercially available interpretation platform increases the costs compared to the use of freely available software, we opted for the former for data security reasons, consistency in the analyses, and to build this project with standards that could be transferred to a clinical laboratory. The hybrid strategy used in DECIPHERD can serve as an intermediate step in establishing autonomous local genomic services for undiagnosed diseases and other genetic conditions in settings of similar limited resources.

As result of the study, we were able to identify a molecular etiology for almost half of the participants across a wide range of clinical phenotypes. This is similar to published data from large studies in European and North American populations [10, 25] and higher than studies in Argentina and Brazil, reporting detection rates of 30–40% [26, 27]. Our approach also identified patients in special situations, such as known dominant conditions presenting in probably recessive forms, and also potential dual diagnosis.

ES had higher diagnostic yield than CES in this study. We explored the latter strategy in the initial phase of the program, given the available local sequencing capacities and published reports showing that proband-only CES could be a cost-effective solution in resource-limited settings [28]. Nevertheless, the use of CES limits the identification of potential novel candidate genes, which can be of special relevance in understudied populations. In addition, and as seen in other admixed populations, de novo variants were a major contributor to the severe phenotypes observed in the patients, highlighting the value of including parental studies [10, 25, 29, 30]. The ES pipeline also allowed detection of both SNVs and CNVs, demonstrating the benefits of a single integrated genomic test for persons with RUD. These observations provide arguments in favor of the recommendation for trio ES analysis to increase diagnostic rates this population [6].

Regarding the inherited variants, three of the eight patients with homozygous findings reported parental consanguinity. This information may have been unknown or undeclared by the rest of the families, or the findings may alternatively suggest founder effects that could be of relevance to the broader Chilean population.

Importantly, almost half of our informative findings have not been previously described in large, publicly available population or clinical databases such as gnomAD and ClinVar. This knowledge gap in local genomic population also impacts the filtering process; the use of an internal database, generated through this and other ongoing projects was crucial for interpretation. This highlights the need to increase diversity of genomic variation studies, since including understudied populations can make substantial contributions to relevant new knowledge, valuable for clinical and other genomic applications [31–33].

The presence of neurologic findings (mainly intellectual disability) was significantly associated with diagnostic yield, while immune dysfunction was negatively associated. The former is similar to the recent findings of Wright et al. [10], but we did not find other statistically significant and clinically relevant associations as shown in their study. This could be due to the small sample size that limits statistical power. It is relevant to note that all probands entering the study due to dysfunction of the immune system had prior negative gene panel testing, while the remaining patients had more heterogeneous pre-entry genetic testing.

Limitations of our study included the relatively small sample size and the small number of samples that underwent head-to-head comparison of results which precluded an in-depth analysis of performance metrics in this implementation process. Pre-inclusion clinical evaluations were very heterogeneous among participants as mentioned above, owing to the different availability of genetic services across the country. Despite DECIPHERD´s efforts to have broad geographic representation and work as a country-wide network, most of the participants came from the Metropolitan Region of Santiago, which has the largest concentration of the population and even disproportionately larger concentration of medical subspecialists [16]. Despite these limitations and potential biases, this work likely represents a “real-world” situation for Chilean RUD patients.

Several next steps are necessary to increase the diagnostic yield and to implement genomic sequencing in the Chilean health care system. First, the study of this understudied population has the potential of identifying variants in novel, unassociated candidate genes. Selected variants in novel candidate genes have been shared in GeneMatcher [34] and connections with colleagues worldwide are underway. We anticipate this may contribute to the discovery of new genetically determined disorders. Second, reanalysis has proven to improve diagnostic yield of genomic testing based on new discoveries and updated bioinformatic tools [25]. Our team is committed to reanalyzing exome results of this cohort after at least one year of initial analysis, and we predict this could also contribute to improving the diagnostic yield. In addition, we plan to analyze secondary findings among the families that consented to receive them, which may lead to additional clinically relevant findings. It would be ideal to also implement other strategies such as genome and/or transcriptome sequencing to further increase diagnostic yield for patients with non-informative or partially informative findings [35–37].

Nevertheless, local implementation of a sequencing pipeline is still hindered by prohibitive costs. If prices drop to cost-efficient levels for LMIC countries, it may be feasible to implement NGS at scale in our country. Sample volume is a pricing factor, particularly in a small country, and therefore collaboration between different areas and centers that use NGS can also facilitate the development of local sequencing centers. Formal and more extensive training of clinical, laboratory and bioinformatics workforce is also crucial, along with certification and accreditation. Performing testing in the country is key for patient access, as the Chilean health system does not provide financial coverage for testing performed abroad.

Another crucial element to consider for decision-makers is the impact of reaching a diagnosis. In parallel to the diagnostic pipeline described in this article, our team is collecting quantitative and qualitative information on the effects of achieving a genetic diagnosis for patients, caregivers, healthcare teams and the healthcare system to contribute with locally pertinent data and insights that can guide the elaboration of national policies.

Our work highlights the feasibility of establishing a program for rare and undiagnosed diseases in a country with limited genomic resources. This is aligned with the World Health Organizations call to advance genomic medicine worldwide [14], and the United Nations Sustainable Development Goals to “leave no one behind” [38]. We expect this work may be useful for other countries in similar situations to develop their own RUD programs.

## Supplementary information

Supplementary Table 1

## Data Availability

The dataset that was generated and/or analyzed as part of this study is available at https://dataview.ncbi.nlm.nih.gov/object/PRJNA1033354?reviewer=k9j1lk2nlqrfp1f4cbv4bi7ma2.
